# Higher blood hematocrit predicts hyperuricemia: a prospective study of 62897 person-years of follow-up

**DOI:** 10.1038/srep13765

**Published:** 2015-09-04

**Authors:** Chao Zeng, Jie Wei, Tuo Yang, Hui Li, Wen-feng Xiao, Wei Luo, Shu-guang Gao, Yu-sheng Li, Yi-lin Xiong, Guang-hua Lei

**Affiliations:** 1 Department of Orthopaedics, Xiangya Hospital, Central South University, Changsha, Hunan Province, China, 410008; 2 Department of Epidemiology and Health Statistics, School of Public Health, Central South University, Changsha, Hunan Province, China, 410008

## Abstract

This prospective study aimed to investigate the relationship between higher hematocrit (Hct) level and hyperuricemia (HU) incidence. A total of 27540 subjects were included. Baseline Hct was classified into four categories based on the quartile distribution of the study population. A cox proportional hazards regression was used to evaluate the risk of HU incidence across the Hct quartiles after adjusting a number of potential confounding factors. Out of the 62897 person-years of follow-up, 2745 new cases of HU were developed. In models adjusted for known risk factors of HU, higher Hct was used to predict HU incidence independently in a graded manner (p = 0.02): compared with subjects in the lowest quartile, subjects in the highest quartile of Hct (hazard ratio = 1.20; 95% confidence interval: 1.03–1.41) were n20% more likely to develop HU. Sensitivity analysis indicated that the hazard ratios increased with the extension of the minimum follow-up interval. When the minimum follow-up interval was restricted to 4 years, subjects in the highest quartile of Hct were 70% more likely to develop HU, compared with the lowest quartile. Higher Hct, a routinely measured inexpensive biomarker was independently associated with the incidence of HU even within the normal range.

Hyperuricemia (HU) has become a major public health issue worldwide because of its high and increasing prevalence in the global context^1^,^2^,^3^, and has long been recognized as a risk factor for the development of gout, diabetes, hypertension, stroke, artherosclerosis, cardiovascular disease, and chronic kidney disease^4^,^5^,^6^,^7^,^8^,^9^. A national survey reported that approximately 21.4% of adults in the US suffered from HU^10^. Meanwhile, the prevalence of HU is ranged from 13% to 25.8% in some Asian countries^11^,^12^,^13^,^14^. Therefore, the early detection, treatment and preventive methods of HU have drawn wide concern from the academic circle, but up to present, the specific pathogenesis of HU has not yet been fully elucidated.

Hematocrit (Hct), the proportion of blood volume occupied by red blood cells, is a major determinant of blood viscosity^15^,^16^. Many previous studies have proved that higher Hct or blood viscosity was positively associated with insulin resistance^17^,^18^,^19^,^20^,^21^,^22^,^23^,^24^,^25^, a physiological condition in which cells fail to respond to the normal actions of hormone insulin. Insulin resistance has been proved to be correlated with higher serum uric acid or HU^26^,^27^,^28^,^29^,^30^,^31^,^32^, although the causal effect between them remained unclear. Considering all these factors, higher Hct may be associated with HU, although it has not yet been explored by any researcher, not to mention the causal relationship. Only a few studies indicated a positive association between Hct and serum uric acid^33^,^34^,^35^. Therefore, the objective of this prospective study was to explore the aforementioned causal relationship based on the following hypothesis: higher blood Hct level at baseline is associated with an increased risk of HU during the follow-up period.

## Methods

### Study population

The study was approved by the ethics committee at Xiangya Hospital, Central South University, and was conducted in accordance with the Protocol of Helsinki. Informed consent in writing was obtained from each subject participating in this study. In the present study, subjects who underwent health examinations between 2007 and 2014 in the Department of Health Examination Center Xiangya Hospital, Central South University in Changsha, Hunan Province, China were recruited. This huge epidemiological prospective study mainly aimed to explore the risk factors (e.g. blood index) of HU and the risk factors of other disease (e.g. urinary calculus) in patients with HU. Routine health checkups are very common in China, because the Chinese government encourage people to take periodic medical examinations. Three of our screen-based cross-sectional studies have been published^36^,^37^,^38^. Subjects who received annual or biennial health examination twice or more (a total of 53984 subjects between 2007 and 2014) were considered to be included in this prospective study. The first health examination was considered as the baseline exposure. Then, 42505 subjects who were free of HU at baseline were identified. Subjects who were younger than 18 years old at baseline (n = 64), or with missing data of Hct level at baseline (n = 4387), or with missing data of uric acid at the subsequent health examination (n = 2594) were excluded. Subjects with missing data of important physical examination results at baseline (n = 7920), such as body mass index (BMI), blood pressure and blood glucose, were also excluded from this study. Eventually, 27540 subjects with the follow-up interval ranged from 1 to 7 years were qualified.

### Baseline measurement

All blood samples were drawn after a 12-hour overnight fast and were kept at 4 °C until analysis. Hct, white blood cell count (WBC), and platelet count were detected by the Beckman Coulter LH750 automated hematology analyzer (Beckman Coulter Inc., Miami, FL, USA). Uric acid, fasting blood glucose (FBS), high-density lipoprotein cholesterol (HDL- cholesterol), low-density lipoprotein cholesterol (LDL- cholesterol), triglyceride, serum creatinine (SCr) and alanine aminotransferase (ALT) were detected by the Beckman Coulter AU 5800 (Beckman Coulter Inc., Brea, CA, USA). Systolic blood pressure (SBP) and diastolic blood pressure (DBP) were measured using an electronic sphygmomanometer. BMI was calculated as weight (kg) divided by height squared (m^2^).

### Ascertainment of incident cases of HU

Subjects who were free of HU at baseline and received a diagnosis of HU at any subsequent health examination were defined as incident HU cases. The end point was the first recorded incidence of HU during the follow-up interval or the end of the study period (November 2014). HU was defined by the UA ≥ 416 mmol/L in male population and ≥360 mmol/L in female population.

### Statistical analysis

The quantitative data were expressed as mean (standard deviation), and the qualitative data were expressed in percentage. Baseline Hct was classified into four categories based on the quartile distribution in the whole sample: <37.4%, 37.4%–40.3%, 40.4%–43.7%, and >43.7%. Differences in continuous data were evaluated by the one-way classification ANOVA (normally distributed data) or the Kruskal-Wallis H test (non-normally distributed data), while differences in qualitative data were assessed by the χ^2^ test. The person-years of follow-up were computed as the time interval from the first health examination (baseline) to the day of clinical diagnosis of HU at a subsequent examination, or the day of the last attended examination. The cumulative curves for the incidence of HU according to baseline Hct quartiles over the follow-up period were used; the Kaplan–Meier method and the log-rank test were used to evaluate the statistical significance of the difference among Hct quartiles. The cox proportional hazards regression was used to evaluate the risk of incident HU across the Hct quartiles. The unadjusted, age-sex-BMI adjusted, and multivariable adjusted hazard ratios (HR), as well as the related 95% confidence intervals (95%CI) were reported respectively. The following confounders from baseline were included in the multivariable model: age, sex (male or female), BMI (≥25 kg/m^2^ or <25 kg/m^2^), WBC, platelet count, LDL cholesterol, HDL cholesterol, triglyceride (log-transformed), ALT (log-transformed), estimated glomerular filtration rate (eGFR), FBS (log-transformed), SBP and DBP. eGFR was calculated by serum creatinine (Scr), sex, and patients’ age. The calculation formula was: 186 × SCr^−1.154^ × age^−0.203^ × 1.210(if black) × 0.742(if female)^39^. These confounding factors were chosen based on some previous studies^40^,^41^,^42^. Tests for linear trends were conducted based on the cox proportional hazards regression using a median variable of Hct level in each category. Sensitivity analysis was conducted by setting different minimum follow-up intervals (2, 3 and 4 years) to examine the risk of HU across the reclassified Hct quartiles. Then, subgroup analysis was conducted after stratifying data by sex. Baseline Hct was reclassified into four categories based on the quartile distribution in the male population: <41.4%, 41.4%–43.5%, 43.6%–45.5%, and >45.5%, and in the female population: <35.7%, 35.7%–37.5%, 37.6%–39.3%, and >39.3%. Analysis was performed separately for male and female subjects. All data were analyzed using SPSS 17.0; p ≤ 0.05 was considered to be statistically significant.

## Results

A total of 27540 subjects involving 62897 person-years of follow-up were included in the present study. The follow-up intervals were ranged from 1 to 7 years (mean 2.78 years). The sample was composed of 14422 males (52.4%) and 13118 females (47.6%), with an average age of 42.08 years (standard deviation 13.52) at baseline. The range of Hct in the study population was from 17.8% to 61.0% (mean 40.4%, median 40.3%). The baseline characteristics of the study population across the quartiles of Hct were shown in Tables 1 and 2. Age, sex ratio, BMI, WBC, platelet count, HDL-cholesterol, LDL-cholesterol, triglyceride, SCr, ALT, FBS, SBP and DBP were significantly different across the Hct quartiles. During the follow-up years, a total of 2745 incident cases (2068 males, 677 females) of HU were identified. The incidence of HU was 43.6 per 1000 person-years in the total sample (62.5 per 1000 person-years in males and 22.7 per 1000 person-years in females).

**Figure 1:**
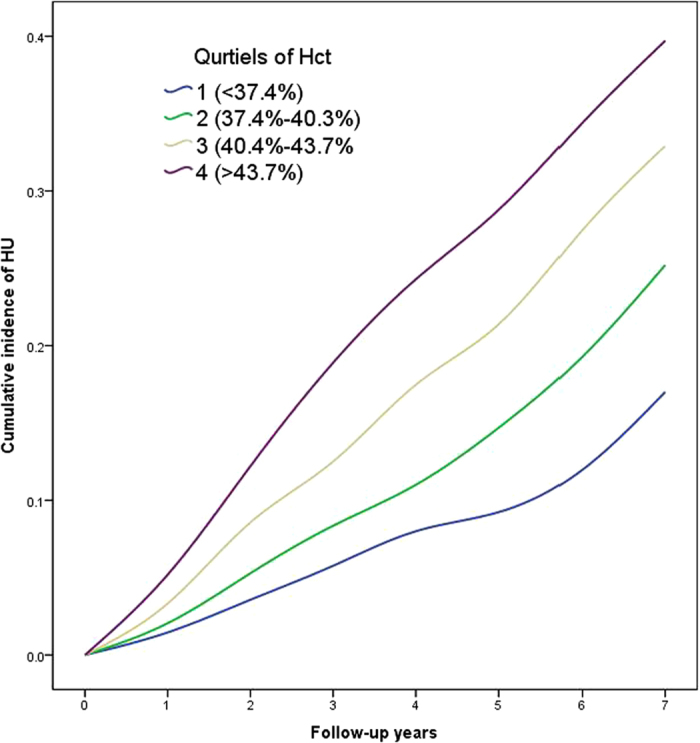
The cumulative curves for the incidence of HU according to Hct quartiles during 7 years of follow-up.

Figure 1 showed the cumulative incidence of HU up to 7 years of follow-up. The curves suggested that subjects in the higher quartiles of Hct exhibited higher incidence of HU, compared with those in the lower quartiles (log-rank test, p = 0.000). Cox proportional hazards regression models were used to examine the risk of HU incidence across the Hct quartiles. The outcomes were shown in Table 3. Unadjusted HRs indicated a strongly increased risk of HU in the second (HR: 1.46, 95%CI: 1.28 to 1.67), third (HR: 2.21, 95%CI: 1.96 to 2.50), and fourth quartile (HR: 3.09, 95%CI: 2.74 to 3.47), compared with the lowest quartile of Hct (p for trend = 0.00). With adjustment of age, sex and BMI, the risk of HU was still significantly increased in the second (HR: 1.16, 95%CI: 1.01 to 1.33), third (HR: 1.20, 95%CI: 1.04 to 1.39) and fourth quartile (HR: 1.44, 95%CI: 1.24 to 1.68) of Hct level, compared with the lowest quartile (p for trend = 0.00). The multivariable adjusted HR (adjusted by age, sex, BMI, WBC, platelet count, LDL cholesterol, HDL cholesterol, triglyceride, ALT, SCr, FBS, SBP and DBP) suggested a significantly increased risk of HU in the highest quartile of Hct level (HR: 1.20, 95%CI: 1.03 to 1.41), compared with the lowest quartile. The p for trend was 0.02. Relative to subjects in the lowest quartile, those in the highest quartile of Hct were 20% more likely to develop HU.

Sensitivity analysis was conducted by restricting different minimum follow-up intervals (2, 3 and 4 years) to examine the risk of HU across the reclassified Hct quartiles (Table 3). With the minimum follow-up interval of 2 years (60418 person-years), the multivariable adjusted HR suggested a significantly increased risk of HU in the highest quartile of Hct level (HR: 1.23, 95%CI: 1.05 to 1.44), compared with the lowest quartile. P for trend was 0.01. With the minimum follow-up interval of 3 years (48818 person-years), the multivariable adjusted HR suggested a significantly increased risk of HU in the highest quartile of Hct level (HR: 1.30, 95%CI: 1.11 to 1.52), compared with the lowest quartile (p for trend = 0.00). Meanwhile, with the minimum follow-up interval of 4 years (37378 person-years), the multivariable adjusted HR also suggested a significantly increased risk of HU in the second (HR: 1.27, 95%CI: 1.11 to 1.46), third (HR: 1.38, 95%CI: 1.18 to 1.61) and the highest quartile of Hct level (HR: 1.70, 95%CI: 1.44 to 2.00), compared with the lowest quartile (p for trend = 0.00).

Subgroup analysis was conducted to assess the risk of HU across different Hct levels in the male and female population respectively (Tables 4 and 5). For male subjects, the unadjusted HRs suggested a significant higher incident HU in the third (HR: 1.13, 95%CI: 1.00 to 1.28) and fourth quartile (HR: 1.36, 95%CI: 1.21 to 1.54), compared with the reference level (p for trend = 0.00). With adjustment of age, sex and BMI, the risk of HU was still significantly increased in the fourth quartile (HR: 1.22, 95%CI: 1.08 to 1.38) of Hct level, compared with the lowest quartile (p for trend = 0.00). However, the multivariable adjusted HR just approached statistical significance (p for trend = 0.09). With the minimum follow-up interval of 3 or 4 years, the multivariable adjusted HRs suggested a significantly increased risk of HU in the highest quartile of Hct level compared with the lowest quartile (p for trend = 0.01 and 0.00, respectively). For female subjects, the unadjusted HR (HR: 1.70, 95%CI: 1.38 to 2.09), the age and BMI adjusted HR (HR: 1.51, 95%CI: 1.23 to 1.86), and the multivariable adjusted HR (HR: 1.26, 95%CI: 1.01 to 1.56) all showed a significantly increased risk in the highest quartile of Hct level, compared with the lowest quartile (p for trend = 0.00, 0.00 and 0.02, respectively).

## Discussion

This prospective study suggested that Hct is an independent predictor of HU. This relationship was graded (p for trend < 0.05) and independent of a wide range of established HU risk factors. Compared with subjects in the lowest quartile, those in the highest quartile of Hct were nearly 30% more likely to develop HU. In addition, the probability increased with the extension of the minimum follow-up interval. For example, when the minimum follow-up interval was restricted to 4 years, subjects in the highest quartile of Hct were 76% more likely to develop HU, compared with the lowest quartile. Therefore, elevated Hct deserves attention as an emerging risk factor for HU. Hct is a marker (type 0) of natural history of HU that correlates with known clinical indices.

In addition to the studies directly suggested a positive association between Hct and serum uric acid^33^,^34^,^35^, some other researches indicated a positive trend between Hct and serum uric acid in a variety of settings, such as screen-based cohorts^42^,^43^, subjects with intact kidney function in community-based cohorts^44^, screen-based cross-sectional survey^45^, slightly reduced glomerular filtration rate^46^, and angiographically near normal coronary arteries^47^, even though the significance of difference remained unclear. The present study was the first one demonstrating that higher Hct level at baseline is associated with an increased risk of HU during the follow-up period.

Some studies suggested that insulin resistance was positively associated with serum uric acid in the screen-based cross-sectional survey^28^, and the normal glucose tolerance and normal fasting glucose subjects^30^. In addition, some other studies directly reported a positive relationship between insulin resistance and HU in a variety of settings, such as nondiabetic subjects with varying degrees of the metabolic syndrome^26^, a cross-sectional survey in young black and white adults^27^, and a community-based sample of Portuguese adults^31^. Based on these direct and indirect evidences, it was speculated that higher Hct might be a risk factor for HU, which has been proved by the present study. However, it is noteworthy that higher serum uric acid or Hu could also possibly contribute to insulin resistance. Carnethon *et al.*^48^ indicated a significantly higher risk of hyperinsulinemia in subjects with increased baseline uric acid level in a prospective study. Similarly, Krishnan *et al.*^49^ revealed that patients with HU are subjects to 1.36 times of risk for developing insulin resistance compared to normal subjects in a 15-year follow-up study. Moreover, the effect of decreased uric acid on insulin resistance was demonstrated by a mice study^50^. These researches implied a possible feedback effect.

**Figure 2:**
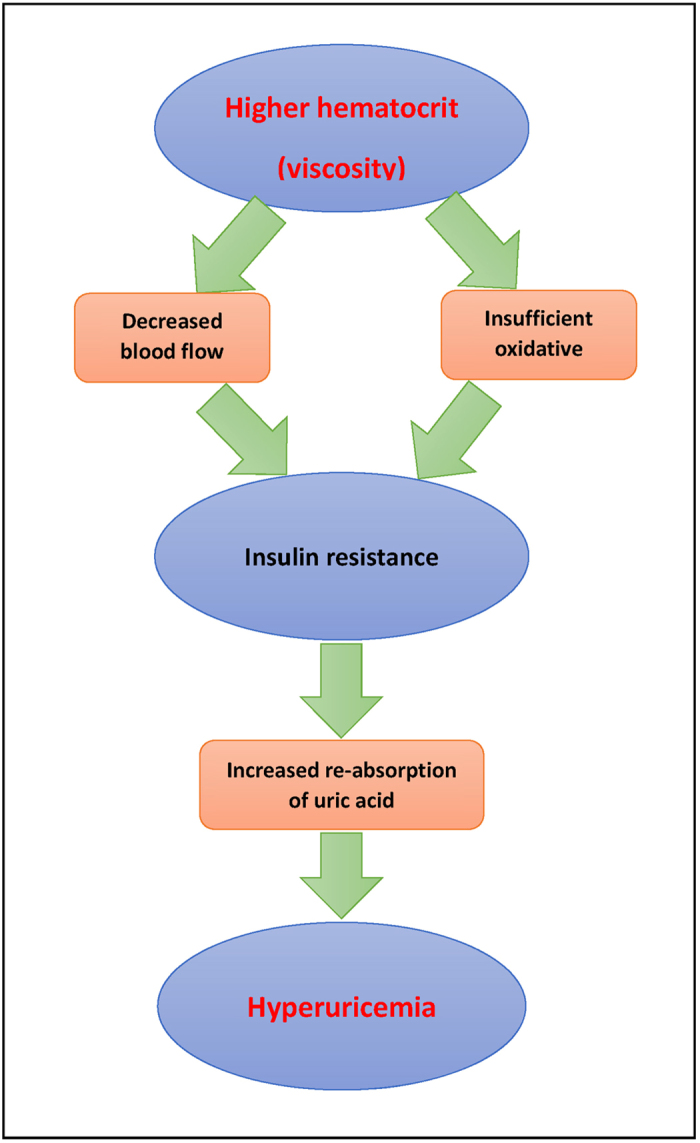
Possible mechanisms of higher Hct serving as the risk factor for the incidence of HU.

Increased blood viscosity may contribute to insulin resistance through two mechanisms: decreased blood flow and insufficient oxidative capacity^51^. According to Poiseuille’s law^52^, blood flow is inversely proportional to the whole blood viscosity. Thus, the increased blood viscosity may result in decreased blood flow, which thereby reduces the delivery of oxygen, glucose and insulin to skeletal muscle^53^,^54^. Under such a circumstance, vasodilatation and increased blood pressure which belong to the compensatory mechanisms could increase blood flow^55^,^56^,^57^. Once the compensatory mechanisms fail to maintain a proper blood flow, the increased blood viscosity should be sufficient to increase the glucose and insulin level^51^. For another side, a large number of studies suggested that insufficient oxidative capacity could be a primary cause of insulin resistance^58^,^59^,^60^,^61^,^62^,^63^,^64^,^65^,^66^, while oxidative capacity might be reduced by increasing blood viscosity through impaired oxygen delivery^51^. Once the compensatory mechanisms fail to fully restore the oxygen delivery, impaired oxidative capacity could increase insulin resistance^51^. Furthermore, the increased insulin level can enhance renal tubular sodium reabsorption, which in turn reduces renal excretion of uric acid^67^,^68^,^69^,^70^. Figure 2 showed the possible mechanism between Hct and HU.

The present study is featured with a number of strengths, such as prospective design, large sample (27540 subjects), high event rates, almost equal distribution between male and female subjects, and effect sizes adjusted for multiple confounding factors. To our best knowledge, this is the first study demonstrating that higher blood Hct is an independent predictor of HU. However, limitations should also be acknowledged. Firstly, although the incidence of HU was high, the follow-up interval (1 to 7 years) was not long enough. However, the value of HR increased with the extension of the minimum follow-up interval, suggesting that the observed association was underestimated. Secondly, the use of uric-acid-lowering drugs during the follow-up was not available, but this would also be expected to weaken the noted observed association. Meanwhile, the percentage of long-term use of uric-acid-lowering drugs was very low (3/3789), which was observed in our previous research^39^. Similarly, the use of antihypertensive, antilipidemic, and antidiabeteic medications was not recorded. It must be admitted that these drugs could have the potential to influence the serum uric acid level. But this would also be expected to weaken the noted observed association in most cases. Thirdly, due to the lack of information on smoking status and alcohol consumption, the influence of these factors on the association between Hct and serum uric acid level could not be determined. Fourthly, differing from the community-based survey conducted in western countries, the present study is a screen-based prospective cohort, which is similar to some other high-quality screen-based studies conducted in Asian countries, such as Japan^71^, Korea^42^ and China^72^. Routine health checkups have become very common in Asia. Fifthly, since estrogenic compounds can enhance the renal urate excretion and increase urate clearance, menopause should have strong impact on serum uric acid level in women, and this could affect the results of the present study, because there are both pre-menopause and post-menopause women in these participants. Sixthly, about one-third of the subjects were excluded from the present study because of many reasons, so there is a possibility that selection bias exist. Lastly, the study subjects were extracted from the Chinese population, so the findings may not be generalizable to other populations.

## Conclusions

The results of this study demonstrated that higher Hct level, even within the normal range, was associated with the incidence of HU, independent of multiple risk factors. Elevated Hct, a routinely measured inexpensive biomarker, deserves attention as an emerging risk factor for HU. Further studies should be conducted to determine whether these findings are reproducible in other populations.

## Additional Information

**How to cite this article**: Zeng, C. *et al.* Higher blood hematocrit predicts hyperuricemia: a prospective study of 62897 person-years of follow-up. *Sci. Rep.* **5**, 13765; doi: 10.1038/srep13765 (2015).

## Figures and Tables

**Table 1:** Baseline characteristics of study population across the quartiles of baseline Hct.

	Quartile of baseline Hct				P*
	1 (lowest)	2	3	4 (highest)	
Median (range)	35.5 (<37.4)	38.8 (37.4–40.3)	42.0 (40.4–43.7)	45.7 (>43.7)	–
No. of subjects	6908	6936	6899	6797	–
Age (years)	41.92 (13.76)	42.18 (14.47)	43.15 (13.69)	41.07 (11.94)	0.00
Male (%)	8.8	27.1	76.0	98.4	0.00
BMI (kg/m^2^)	21.83 (2.75)	22.45 (3.03)	23.61 (3.09)	24.56 (3.03)	0.00
WBC (×10^9^/L)	5.79 (1.48)	6.11 (1.50)	6.41 (1.57)	6.77 (1.67)	0.00
Platelet count (×10^9^/L)	193.72 (57.54)	195.22 (51.27)	191.37 (52.13)	193.48 (49.60)	0.00
HDL-cholesterol (mmol/L)	1.53 (0.34)	1.49 (0.35)	1.36 (0.33)	1.27 (0.29)	0.00
LDL-cholesterol (mmol/L)	2.47 (0.75)	2.60 (0.77)	2.66 (0.80)	2.80 (0.84)	0.00
Triglyceride (mmol/L)	0.99 (0.82)	1.13 (0.97)	1.51 (1.49)	1.75 (1.52)	0.00
SCr (μmol/L)	66.97 (21.2)	69.78 (18.92)	81.31 (17.16)	85.88 (13.46)	0.00
ALT (U/L)	17.82 (12.74)	20.31 (14.35)	25.99 (22.15)	32.17 (23.87)	0.00
FBS (mmol/L)	4.98 (0.69)	5.07 (0.88)	5.19 (1.09)	5.23 (1.33)	0.00
SBP (mmHg)	114.76 (16.41)	117.89 (17.23)	122.33 (16.47)	123.80 (15.67)	0.00
DBP (mmHg)	70.88 (10.25)	73.65 (10.63)	77.40 (11.11)	79.80 (11.34)	0.00

Hct, hematocrit; BMI, body mass index; WBC, white blood cell; HDL-cholesterol, high density lipoprotein cholesterol; LDL-cholesterol, low density lipoprotein cholesterol; SCr, serum creatinine; ALT, alanine aminotransferase; FBS, fasting blood glucose; SBP, systolic blood pressure; DBP, diastolic pressure.

Data are mean (Standard Deviation), unless otherwise indicated.

*P values are for test of difference across all quartiles of baseline Hct.

**Table 2:** Baseline characteristics of study population across the quartiles of baseline Hct in gender subgroup.

	Quartile of baseline Hct				P*
	1 (lowest)	2	3	4 (highest)	
Male subgroup					
Median (range)	39.8(<41.4)	42.5(41.4–43.5)	44.5(43.6–45.5)	46.9(>45.5)	–
No. of subjects	3669	3713	3461	3579	–
age (years)	48.35(16.25)	43.78(13.3)	42.09(12.42)	40.19(11.47)	0.00
BMI (kg/m^2^)	23.68(3)	24.07(2.97)	24.4(2.95)	24.71(3.07)	0.00
WBC (×10^9^/L)	6.2(1.57)	6.48(1.6)	6.62(1.62)	6.9(1.69)	0.00
Platelet count (×10^9^/L)	181.02(53.87)	188.37(51.04)	191.67(48.72)	194.29(50.2)	0.00
HDL-cholesterol (mmol/L)	1.31(0.32)	1.29(0.31)	1.27(0.29)	1.26(0.3)	0.00
LDL-cholesterol (mmol/L)	2.58(0.8)	2.66(0.83)	2.75(0.83)	2.83(0.85)	0.00
Triglyceride (mmol/L)	1.51(1.34)	1.68(1.71)	1.7(1.48)	1.8(1.57)	0.00
SCr (μmol/L)	90.08(26.23)	87.33(13.52)	86.19(13.3)	86.34(12.93)	0.00
eGFR (ml/min/1.73 m^2^)	87.62(18.71)	91.28(18.04)	93.34(19.15)	93.7(17.62)	0.00
ALT (U/L)	25.31(19.79)	28.07(22.69)	30.93(28.51)	33.5(22.57)	0.00
FBS (mmol/L)	5.25(1.12)	5.21(1.12)	5.21(1.19)	5.24(1.44)	0.00
SBP (mmHg)	125.43(17.88)	123.38(15.76)	123.59(15.45)	124.08(15.76)	0.00
DBP (mmHg)	77.17(11.34)	78.31(11.03)	79.17(11.17)	80.39(11.39)	0.00
Female subgroup					
Median (range)	34.3(<35.7)	36.6(35.7–37.5)	38.4(37.6–39.3)	40.5(>39.3)	–
No. of subjects	3401	3276	3276	3165	–
age (years)	41.07(12.64)	40.38(12.87)	39.65(12.76)	40.33(13.51)	0.00
BMI (kg/m^2^)	21.65(2.65)	21.75(2.71)	21.92(2.89)	22.27(3.08)	0.00
WBC (×10^9^/L)	5.67(1.46)	5.88(1.42)	6.07(1.49)	6.25(1.5)	0.00
Platelet count (×10^9^/L)	195.86(59.91)	195.86(52.24)	200.14(50.22)	202.8(51.93)	0.00
HDL-cholesterol (mmol/L)	1.55(0.33)	1.55(0.33)	1.56(0.33)	1.56(0.33)	0.00
LDL-cholesterol (mmol/L)	2.42(0.73)	2.52(0.76)	2.6(0.75)	2.67(0.78)	0.00
Triglyceride (mmol/L)	0.94(0.7)	0.97(0.82)	0.96(0.65)	1.05(0.81)	0.00
SCr (μmol/L)	65.21(12.82)	63.18(12.7)	62.42(11.99)	61.9(12.05)	0.00
eGFR (ml/min/1.73 m^2^)	98.13(25.27)	102.47(27.23)	103.83(26.28)	104.58(26.33)	0.00
ALT (U/L)	16.94(10.38)	17.64(10.5)	18.14(11.07)	19.95(12.67)	0.00
FBS (mmol/L)	4.95(0.64)	4.96(0.6)	4.99(0.76)	5.08(0.93)	0.00
SBP (mmHg)	113.33(15.57)	113.83(15.55)	114.54(15.66)	117.59(17.24)	0.00
DBP (mmHg)	69.89(9.84)	70.91(9.92)	72.05(9.96)	74.3(10.8)	0.00

Hct, hematocrit; BMI, body mass index; WBC, white blood cell; HDL-cholesterol, high density lipoprotein cholesterol; LDL-cholesterol, low density lipoprotein cholesterol; SCr, serum creatinine; eGFR, estimated glomerular filtration rate; ALT, alanine aminotransferase; FBS, fasting blood glucose; SBP, systolic blood pressure; DBP, diastolic pressure.

Data are mean (Standard Deviation), unless otherwise indicated.

*P values are for test of difference across all quartiles of baseline Hct.

**Table 3:** The HRs of incident HU across the quartiles of Hct.

	Quartile of baseline Hct				P for trend
	1(lowest)	2	3	4(highest)	
Minimum follow-up = 1 year (62897 person-years)					
Median (range)	35.5(<37.4)	38.8(37.4–40.3)	42.0(40.4–43.7)	45.7(>43.7)	–
No. of subjects	6908	6936	6899	6797	–
Person-years of follow-up	16815	16028	15856.5	14197.5	–
Incidence of HU (per 1000 person-years)	22.8	33.6	50.8	71.7	–
Unadjusted HR (95%CI)	1.00(reference)	1.46(1.28, 1.67)	2.21(1.96, 2.50)	3.09(2.74, 3.47)	0.00
P value	–	0.00	0.00	0.00	–
Age, sex, BMI adjusted HR (95%CI)	1.00(reference)	1.16(1.01, 1.33)	1.20(1.04, 1.39)	1.44(1.24, 1.68)	0.00
P value	–	0.03	0.02	0.00	–
Multivariable adjusted HR (95%CI)	1.00(reference)	1.10(0.96, 1.26)	1.08(0.93, 1.25)	1.20(1.03, 1.41)	0.02
P value	–	0.18	0.34	0.02	–
Minimum follow-up = 2 years (60418 person-years)					
Multivariable adjusted HR (95%CI)	1.00(reference)	1.10(0.96, 1.26)	1.09(0.94, 1.26)	1.23(1.05, 1.44)	0.01
P value	–	1.77	0.26	0.01	–
Minimum follow-up = 3 years (48818 person-years)					
Multivariable adjusted HR (95%CI)	1.00(reference)	1.12(0.98, 1.28)	1.13(0.97, 1.32)	1.30(1.11, 1.52)	0.00
P value	–	0.10	0.11	0.00	
Minimum follow-up = 4 years (37378 person-years)					
Multivariable adjusted HR (95%CI)	1.00(reference)	1.27(1.11, 1.46)	1.38(1.18, 1.61)	1.70(1.44, 2.00)	0.00
P value	–	0.00	0.00	0.00	–

Hct, hematocrit; BMI, body mass index; No., number; HR, hazard ratio.

Multivariable adjusted model included: age, sex (male or female), BMI (≥25 or <25), white blood cell count, platelet count, low-density lipoprotein cholesterol, high-density lipoprotein cholesterol, triglyceride (log-transformed), alanine aminotransferase (log-transformed), Estimated glomerular filtration rate (eGFR), fasting blood glucose (log-transformed), systolic pressure and diastolic pressure.

**Table 4:** The HRs of incident HU across the quartiles of Hct in male population.

	Quartile of baseline Hct				P for trend
	1(lowest)	2	3	4(highest)	
Minimum follow-up = 1 year (33110 person-years)					
Median (range)	39.8(<41.4)	42.5(41.4–43.5)	44.5(43.6–45.5)	46.9(>45.5)	–
No. of subjects	3669	3713	3461	3579	–
Person-years of follow-up	9422.5	8881.5	7727.5	7078.5	–
Incidence of HU (per 1000 person-years)	55.2	56.1	63.9	78.5	–
Unadjusted HR (95%CI)	1.00(reference)	1.01(0.89, 1.14)	1.13(1.00, 1.28)	1.36(1.21, 1.54)	0.00
P value	–	0.93	0.05	0.00	
Age, BMI adjusted HR (95%CI)	1.00(reference)	0.95(0.84, 1.08)	1.04(0.92, 1.18)	1.22(1.08, 1.38)	0.00
P value	–	0.43	0.57	0.00	–
Multivariable adjusted HR (95%CI)	1.00(reference)	0.92(0.81, 1.04)	0.96(0.85, 1.10)	1.11(0.98, 1.26)	0.09
P value	–	0.17	0.57	0.11	–
Minimum follow-up = 2 years (31814 person-years)					
Multivariable adjusted HR (95%CI)	1.00(reference)	0.90(0.79, 1.02)	0.98(0.86, 1.11)	1.13(0.99, 1.28)	0.04
P value		0.10	0.71	0.07	
Minimum follow-up = 3 years (26441 person-years)					
Multivariable adjusted HR (95%CI)	1.00(reference)	0.87(0.77, 0.99)	0.96(0.88, 1.13)	1.15(1.02, 1.31)	0.01
P value	–	0.04	0.96	0.03	
Minimum follow-up = 4 years (20756 person-years)					
Multivariable adjusted HR (95%CI)	1.00(reference)	0.94(0.83, 1.08)	1.09(0.95, 1.24)	1.40(1.23, 1.59)	0.00
P value	–	0.40	0.22	0.00	–

Hct, hematocrit; BMI, body mass index; No., number; HR, hazard ratio.

Multivariable adjusted model included: age, sex (male or female), BMI (≥25 or <25), white blood cell count, platelet count, low-density lipoprotein cholesterol, high-density lipoprotein cholesterol, triglyceride (log-transformed), alanine aminotransferase (log-transformed), Estimated glomerular filtration rate (eGFR), fasting blood glucose (log-transformed), systolic pressure and diastolic pressure.

**Table 5:** The HRs of incident HU across the quartiles of Hct in female population.

	Quartile of baseline Hct				P for trend
	1(lowest)	2	3	4(highest)	
Minimum follow-up = 1 year (29787 person-years)					
Median (range)	34.3(<35.7)	36.6(35.7–37.5)	38.4(37.6–39.3)	40.5(>39.3)	–
No. of subjects	3401	3276	3276	3165	–
Person-years of follow-up	8359.5	7700	7248	6479.5	–
Incidence of HU (per 1000 person-years)	18.9	18.7	23.2	31.9	–
Unadjusted HR (95%CI)	1.00(reference)	0.99(0.79, 1.24)	1.23(0.99, 1.53)	1.70(1.38, 2.09)	0.00
P value		0.92	0.06	0.00	
Age, sex, BMI adjusted HR (95%CI)	1.00(reference)	1.00(0.80, 1.26)	1.20(0.96, 1.49)	1.51(1.23, 1.86)	0.00
P value	–	0.98	0.11	0.00	–
Multivariable adjusted HR (95%CI)	1.00(reference)	0.94(0.75, 1.18)	1.08(0.87, 1.35)	1.26(1.01, 1.56)	0.02
P value	–	0.58	0.48	0.04	–
Minimum follow-up = 2 years (28603.5 person-years)					
Multivariable adjusted HR (95%CI)	1.00(reference)	0.92(0.73, 1.16)	1.09(0.88, 1.36)	1.25(1.01, 1.55)	0.02
P value	–	0.50	0.42	0.04	
Minimum follow-up = 3 years (22377 person-years)					
Multivariable adjusted HR (95%CI)	1.00(reference)	0.97(0.77, 1.23)	1.10(0.87, 1.38)	1.35(1.08, 1.68)	0.00
P value	–	0.83	0.44	0.01	
Minimum follow-up = 4 years (16622 person-years)					
Multivariable adjusted HR (95%CI)	1.00(reference)	1.02(0.80, 1.31)	1.33(1.05, 1.67)	1.61(1.29, 2.02)	0.00
P value	–	0.88	0.02	0.00	

Hct, hematocrit; BMI, body mass index; No., number; HR, hazard ratio.

Multivariable adjusted model included: age, sex (male or female), BMI (≥25 or <25), white blood cell count, platelet count, low-density lipoprotein cholesterol, high-density lipoprotein cholesterol, triglyceride (log-transformed), alanine aminotransferase (log-transformed), Estimated glomerular filtration rate (eGFR), fasting blood glucose (log-transformed), systolic pressure and diastolic pressure.
